# Dr. Davies on Mercury

**Published:** 1821-03-01

**Authors:** 


*An Essay on Mercury; wherein are presented Formulæ for some Preparations of this Metal, including Practical Remarks on the safest and most effectual Methods of administering them, for the Cure of Liver Complaints, Dropsies, Syphilis, and other formidable Diseases incident to the Human Frame; being the Result of long Experience and diligent Observation.* By David Davies, M.D. Member of the Royal Colleges of Physicians and Surgeons, London; and Senior Surgeon to St. Peter's Hospital, Bristol. 8vo. London, pp. 35, 1820.

"Nam ne agricolam quidem aut gubernatorem disputatione, sed usu fieri.—*Celsus*."

Although the current of abuse has run strongly against Mercury in the writings of some modern reformers, and although there was some foundation for the reprehension of the abuses of this powerful medicine in the hands of ignorance and rashness; yet, among all sober-minded practitioners, the medicine holds its original rank, and is more depended on, in a great variety of diseases, than any other article of the pharmacopœia. Every attempt, therefore, which is honestly made, and candidly imparted, for the purpose of pointing out the safest mode of administering this valuable remedy, and preserving the constitution from the effect of mismanagement, should be received with thanks by the profession, although no brilliant discovery be made, nor specious theory advanced.

Our author, who is a veteran in the art, and has had thirty or forty years' hospital as well private practice to confirm or refute preconceived opinions, is convinced, from long observation, "that mercury, introduced cautiously into the system by inunction, more certainly removes disease than by the internal exhibition of it;—also that mercury eradicates diseases more certainly and more safely, when not allowed to pass off by the salivary glands, than when salivation is produced."

"Much has been said and written by theorists on the cure of Syphilis without the aid of Mercury. I feel it therefore an imperious duty to caution the credulous and uninformed against a reliance on the anti-syphilitick power of any other medicine yet discovered. I do not, however, mean to say that the constitution, impaired by disease or by the improper administration of Mercury, may not occasionally derive benefit from the use of other remedies; among which might be named Iron, Sarsaparilla, Bark, Guaiacum, Nitrick Acids, and other tonicks." P. viii.

Dr. Davies thinks it must be allowed that, for internal use, the less mercury (quicksilver) be changed by fire, and the less it contains of mineral or vegetable acids (provided it be sufficiently oxydated to produce the proper effects on the constitution,) the better it will be fitted for eradicating diseases. Under this impression, he offers the following formulæ for milder and safer preparations of mercury than those in common use.

CONFECTIO HYDRARGYRI.

"℞ Hydrargyri purificati,

Mannæ optimæ,* partes æquales,

Tere Hydrargyrum cum Mannâ donec globuli visum fugerint ut fiat Confectio.

PULVIS HYDRARGYRI.

"℞ Confectionis Hydrargyri,
Pulvis Glychyrrhizæ, partes æquales,
Mft. Pulvis signatur Pulvis Hydrargyri.

SOLUTIO HYDRARGYRI.

"℞ Confectionis Hydrargyri, grana triginta duo,
Mucillaginis gummi acaciæ, fluidunciam,
Syrupi, fluidrachmas sex,
Aquæ Cinnamomi, fluidrachmas duas,
Signatur solutio Hydrargyri." 8.

We have often exhibited the pil. hydrargyri in *draughts*, when a repugnance was expressed against pills, and that without any decomposition of the mass. The above formulæ, therefore, draught and powder, may be useful in practice.

When mercury, in the above mild form, is introduced so as to produce a brackish taste in the mouth, swelling of the gums, with a gentle flow of viscid acrid saliva, then, our author thinks the boundary is reached, beyond which the medicine should not be administered, particularly to females and children.

We cannot follow our author into the theory of the *modus operandi* of mercury on the human frame. The subject is involved in great obscurity, and we can only merely mark the phenomena produced, without attempting to explain the *quo modo*, as yet.

Our author prefers the confectio hydrargyri to the officinal pilula hydrargyri of the shops, as agreeing better with

* Molasses or treacle may, I think, be advantageously substituted for the manna; as the former does not undergo decomposition.

the stomach and bowels, and being more uniform in its operation. In functional disorders of the digestive organs, this form is perhaps the best, taken in doses of four to eight grains at night, and a mild purgative the following morning, repeated once or twice a week, and prescribing rhubarb, columba, or carbonate of iron on the intermediate days, with exercise on horseback in the open air.

Dr. Davies lays down a code of instructions for the proper administration of a mercurial course.

We can hardly agree with our author that even "in recent and slight cases of syphilis," the patients may pursue their accustomed avocations, and keep to their usual diet, while taking mercury. We are convinced, that where mercury is at all necessary, low diet and confinement within doors are highly judicious, if not absolutely necessary restrictions. When patients labour under formidable stages of the disease, our author considers the following regulations to be necessary. 1st, The tepid bath—2d, An aperient combined with a few grains of calomel—3d, Flannel dress —4th. Regulated temperature to about 64°—5th. Diluting drink, and milk diet, with arrow-root or wheaten flower—6. Mercurial frictions carefully, but steadily, pursued till the cure is effected, without inducing violent ptyalism.

"In conclusion," says our author, "an anxiety to serve mankind, and a strong conviction in my mind, deduced from extensive practice, diligent observation, and an experience of forty years, impel me to add that this Confection, combined with Hyoscyamus, Myrrh, or Carbonate of Iron, has by me been found a most valuable remedy in Phthisis pulmonalis and Scrophula; united with tonicks, purgatives, and diureticks, of excellent use in Dropsies; after local bleeding by Leeches, joined to appropriate remedies, very beneficial in dispersing indurated Tumors in the female breast, the prostate and other indurated glands. Lastly, combined with Tartarized Antimony, or with Antimonial Powder, it forms one of the best febrifuge remedies, suited to almost every case of Pyrexia." 33, 34.

---

